# Intracranial hemorrhage with concurrent aortic dissection

**DOI:** 10.1016/j.radcr.2022.09.058

**Published:** 2022-10-27

**Authors:** Amro Abdelrahman, Moayad Elgassim, Anas M. Babiker, Waseem Umer, Amina Ahmed, Mohamed Elgassim

**Affiliations:** a Hamad Medical Corporation, P.O. 3050, Doha, Qatar; b Taylors University School of Medicine, Subang Jaya, Selangor Malaysia

**Keywords:** Intracranial hemorrhage, Aortic dissection, CT scan, ICH

## Abstract

Aortic dissection is a rare yet lethal condition that is often missed. Presenting symptoms often include chest pain, abdominal pain, or loss of consciousness. Rarely is it asymptomatic, and the concurring symptoms may draw attention away from the dissection. We present a case of a 51-year-old male who presented to the emergency department with sudden onset of slurred speech and right-sided weakness. The CT scan showed a basal ganglia hemorrhage. However, during the scan, the radiologist incidentally found some aortic changes and recommended an ECG-gated CT scan of the thorax, which revealed a Stanford type B aortic dissection. We aim to shed light on patients presenting with neurological findings and conclude to have aortic dissection to increase awareness and facilitate rapid diagnosis and treatment.

## Background

The primary function of the aorta is transporting oxygenated blood from the heart to the organs. Specialized receptors in the ascending aorta and the aortic arch play an essential role in controlling the interaction between systemic vascular resistance and the heart rate. "Acute Aortic Syndrome" is a spectrum of pathologies that can result in aortic dysfunction and lead to death, including acute aortic dissection, intramural hematoma, penetrating aortic ulcer, and ruptured thoracic aortic aneurysm [1].

At least 30 cases per million people are predicted to occur annually. Without treatment, acute type A aortic dissection is said to have a mortality rate of about 1% per hour at first, with half of the patients expected to die by the third day and nearly 80% by the end of the second week. In comparison, the mortality rate in acute type B aortic dissection is lower; however, it remains significant, accounting for a 10% mortality rate at 30 days and 70% or higher in the highest-risk groups [2].

The disease is linked with many risk factors that can be inherited or acquired. Acquired risk factors are hypertension, old age, pregnancy, and trauma. Conditions such as Ehler-Danlos syndrome, Marfan syndrome, and Loeys-Diez syndrome are considered inherited risk factors for aortic dissection in young people [3].

## Case presentation

A 51-year-old gentleman with a past medical history of type 2 diabetes mellitus and hypertension presented to the hospital with a one-day history of sudden onset slurred speech and right-sided weakness. The patient was in his usual state of health when he developed this event. He works in building construction. He denied any smoking or alcohol intake.

**Fig. 1:**
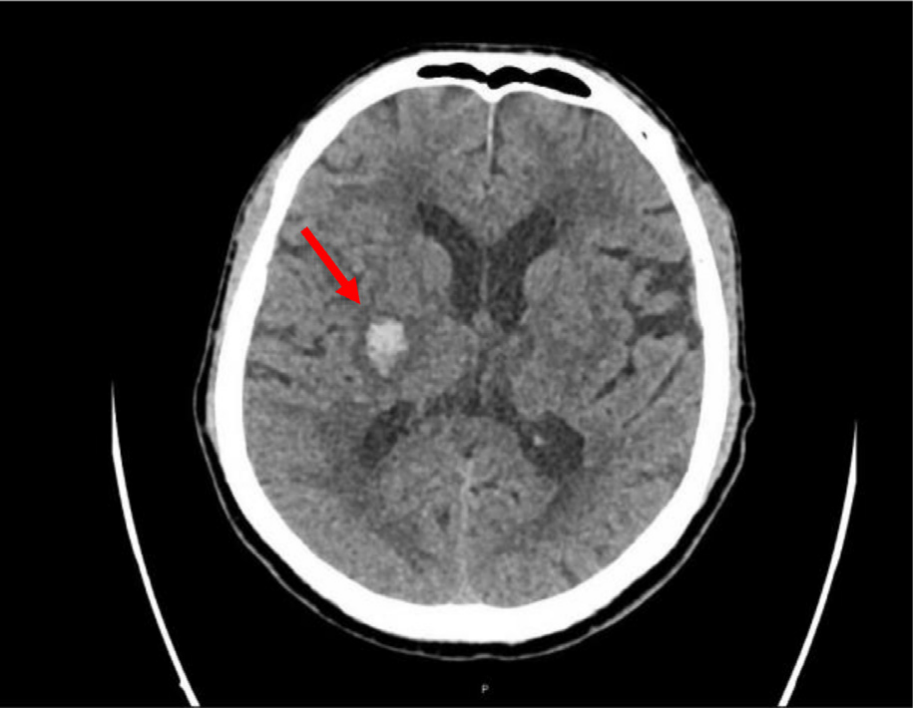
Unenhanced CT brain findings: [Red arrow] right basal ganglia hyper-density suggestive of acute parenchymal bleed measuring approximately 23 × 16 × 12 mm. Left basal ganglia Ill-defined hypodensity likely represents sequela of old insult. Bilateral periventricular/white matter hypodensities suggestive of microangiopathic changes. No significant midline shift or hydrocephalus. Preserved basal cisterns.

On physical examination, the patient was afebrile, his respiratory rate was 19, and oxygen saturation was equal to 95% on room air. The patient was also hypertensive, with a blood pressure of 196/106. His Glasgow Coma Scale (CGS) was 15/15. An apparent right-side weakness involving upper and lower limbs was noted. Chest, abdomen, and legs examinations were all unremarkable. An urgent computed (CT) scan of the head showed right basal ganglia hyperdensity suggestive of acute parenchymal bleed measuring approximately 23 × 16 × 12 mm (Fig. 1).

**Fig. 2:**
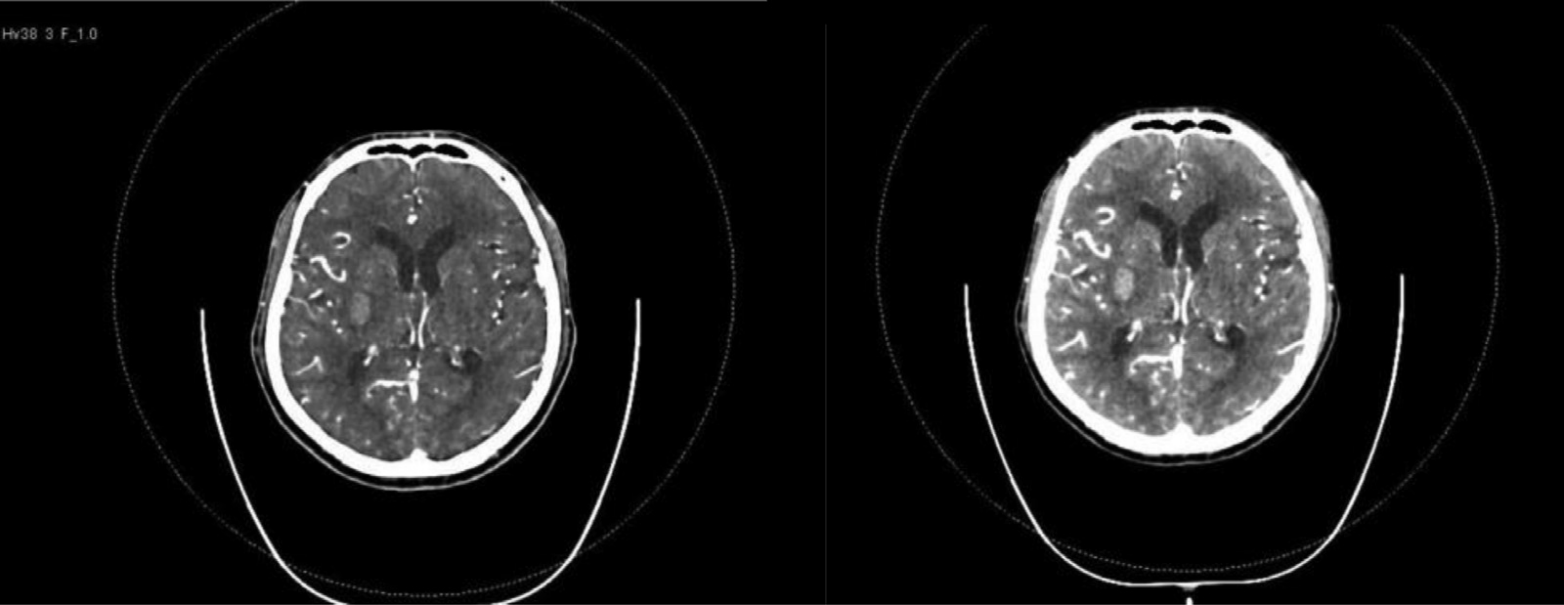
CT intracranial angiography (CTA) findings: atheromatous calcified plaques were noted at bilateral internal carotid arteries. Bilateral internal carotids, MCAs and ACAs, show adequate caliber and contrast opacification. Non-opacification for a small segment at the origin of the right vertebral artery shows diffuse narrow caliber compared to the left. The terminal part of the left vertebral artery shows a small segment of significant stenosis. The basilar artery and bilateral PCAs appear grossly unremarkable. No evidence of intracranial aneurysm or vascular malformation.

**Fig. 3:**
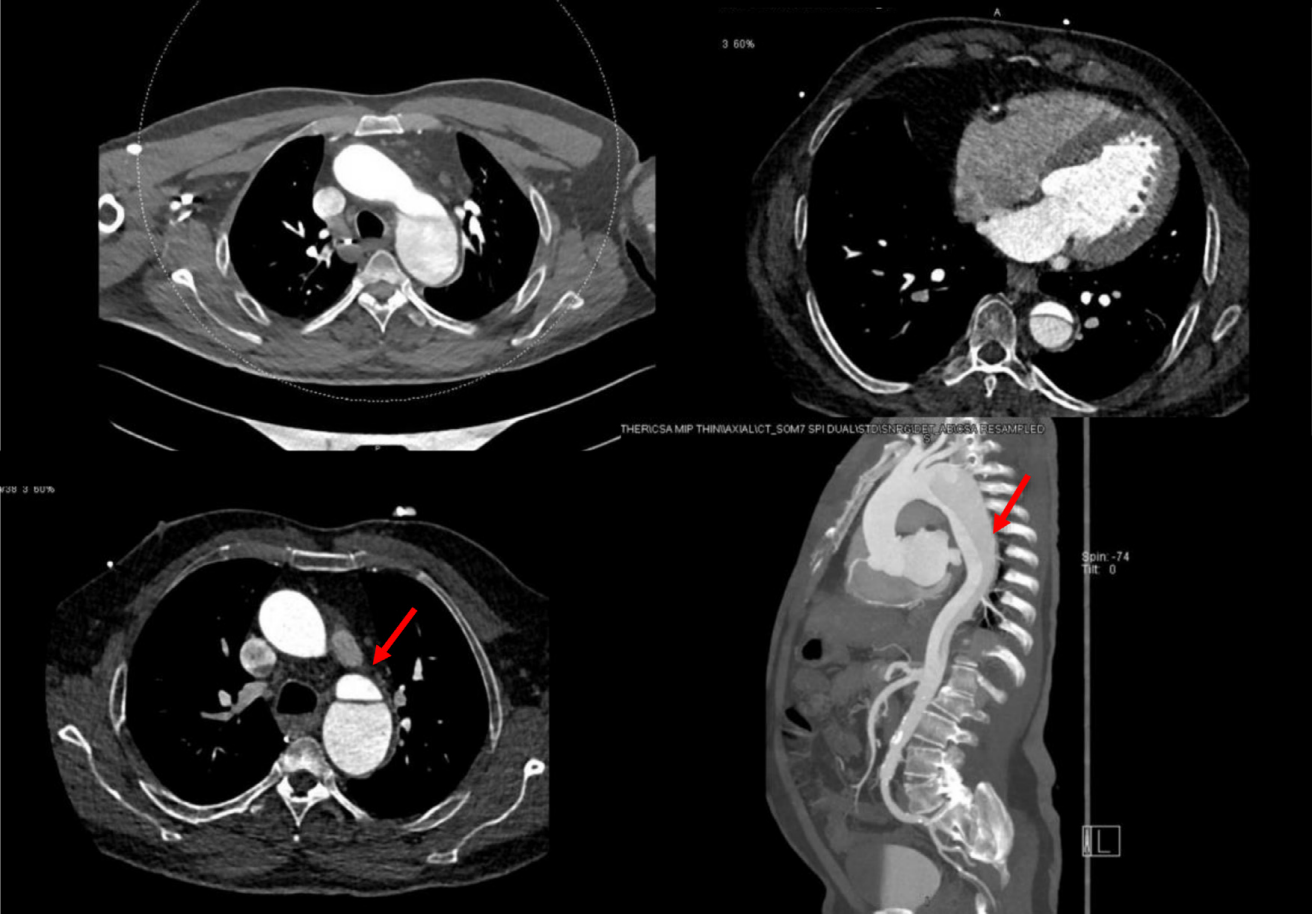
ECG-gated CT aortogram findings: [Red arrow] aortic dissection with flap seen distal to the origin of the left subclavian artery and extending to the level of the celiac trunk. (Stanford type B). Adequate contrast opacification of the major branches of the aorta. Incidentally seen stenosis at the origin of the left main renal artery. Incidentally seen wedging of the L1 vertebral body and partial bony fusion with the vertebral body of T12 (sequela of old injury).

In addition to a CT scan of the head, the patient was evaluated for intracranial hemorrhage by computed tomography angiography (Fig. 2). During the evaluation, the radiologist noticed some aortic changes in the study and discussed it with the referring physician. The decision for a gated CT scan of the thorax was decided. The study showed aortic dissection with the flap seen just distal to the origin of the left subclavian artery and extending to the level of the celiac trunk (Stanford type B aortic dissection) (Fig. 3). The patient was admitted to the medical intensive care unit as his Glasgow Coma Scale (GCS) dropped significantly and intubation and sedation were needed to secure his airways.

The neurology department recommended blood pressure control. Because the aortic dissection is distant to the origin of the left subclavian artery and continues to the level of the celiac trunk, the vascular surgery department was consulted and recommended symptomatic treatment and blood pressure control as there is no surgical intervention needed for this case of type B aortic dissection. With a tracheostomy tube in place, the patient's health improved, and he was transferred to the medical floor.

## Discussion

Aortic dissection is an uncommon disorder, and it usually presents as chest or back pain with a catastrophic illness that can lead to hemodynamic instability. The primary event is tearing aortic intima and degeneration of aortic media with or without medial cystic necrosis.

One of the most widely used classification systems for aortic dissection, known as The Stanford System, classifies dissections that involve the ascending aorta as type A, regardless of the site of the primary intimal tear; all other dissections are classified as type B [4,5].

In our patient, he presented with neurological symptoms related to intracranial hemorrhage, there was no chest or back pain, and the radiologist's diagnosis of aortic dissection was incidental, As type B aortic dissection. A recent study investigated almost 400 patients with type B aortic dissection. It reports that most patients were male (71%), and their average age was around 65 years. As the age advances above 70 years old, the number of cases decreases by 42%. The incidence of total number of cases in all age groups is estimated to be 3 cases per 100,000 patients. The most common risk factors are Hypertension (HTN) (80%) and atherosclerosis (38%) [6].

Age groups and risk factors are essential when cardiovascular diseases are suspected; however, in our case, the patient was relatively young with neurological symptoms as a chief complaint, and he did not experience the classic symptoms related to aortic dissection (chest pain/back pain) or cardiovascular system.

A study showed that about 10% of patients presented with painless aortic dissection and did not show any pulse deficits [7].

In a previous study on patients with thoracic aortic dissection, severe pain is 90% pooled sensitive [8]. One study also reported an aortic dissection with an initial neurologic deficit due to ischemic stroke followed by severe abdominal pain [9], unlike our patient, who did not experience any pain and his neurological symptoms were due to hemorrhagic stroke.

In a retrospective study of over 1000 patients with aortic dissection, 85% experienced the typical symptoms of chest pain or back pain, 90% experienced pain acuity, 49% presented with hypertension with high systolic blood pressure equal to or above 150 mm Hg, and 12% presented with neurological manifestations [7]. Our patient presented initially with right-sided weakness, slurred speech, high blood pressure measuring 196/106 mm Hg, and a painless aortic dissection observed during a CT scan.

In the 1970s and 1980s, Retrograde aortography was the gold standard for diagnosing aortic dissection, but it has been shadowed by cross-sectional imaging, which carries a better safety profile and is more efficacious [10].

Multidetector computed tomography angiography is the first line in investigating patients suspected of aortic dissection. The European Society of Cardiology states this as it allows the assessment of the extent of the dissection, the involvement of the aortic side branches, as well as the relative caliber of the true and false lumens. These factors are relevant to endovascular or open surgery planning [11].

The disadvantages of CT angiography include exposure to ionizing radiation, the inability to assess functional aortic insufficiency, and the use of possibly nephrotoxic contrast media.

Even though chest radiography and electrocardiography are commonly ordered in the emergency setting, they do not exclude or establish the diagnosis of an aortic aneurysm dissection [12].

Our patient's CT head suggests acute parenchymal bleed, which displayed a right basal ganglia hyperdensity. The CT angiography and head indicated aortic changes, prompting the use of a gated CT thorax. The results of the gated CT thorax showed aortic dissection. The flap was distal to the left subclavian artery origin and extended to the celiac trunk level. It was classified as Stanford type B.

The patient suddenly collapsed during the CT scan and was immediately admitted to the ICU for intubation and mechanical ventilation. After consulting with the neurology and vascular surgery department, he was considered a case of uncomplicated type B aortic dissection, and he was given Isosorbide dinitrate (ISDN) infusion, esmolol infusion, and hydralazine to control his blood pressure.

According to the recent guidelines of The Society of Thoracic Surgeons/American Association for Thoracic Surgery, patients with acute/subacute uncomplicated type B aortic dissection could be treated medically, and some cases may be considered for prophylactic thoracic endovascular aortic repair (TEVAR) in order to decrease the risk of late-onset complications (recurrence/death).

Patients with uncomplicated type B aortic dissections may be treated medically. However, some cases may be considered for prophylactic TEVAR to reduce the risk of late-onset complications (recurrence/death), according to the Society of Thoracic Surgeons/American Association for Thoracic Surgery's recent guidelines.

Those with acute complicated type B aortic dissection should undergo TEVAR. If TEVAR obstructs the left subclavian artery circulation, this will increase the risk of spinal cord ischemia; in this case, the revascularization of the left subclavian artery should be done. Patients with chronic type B aortic dissection and evidence of aneurysmal aortic degeneration are excellent candidates for intervention through TEVAR or open surgical repair, depending on their comorbidities.

The recent guidelines also recommend open surgical repair for patients with connective tissue disorders who have type B aortic dissection and failed the medical treatment [13].

## Conclusion

Aortic dissection is a rare but life-threatening condition that requires prompt diagnosis and management. Our patient survived the acute dissection, but unfortunately, his neurological outcome was poor, requiring prolonged hospitalization and weaning from mechanical ventilation.

## Patient consent

Written informed consent was obtained from the patient to publish this report in accordance with the journal's patient consent policy.
